# Tex19 and Sectm1 concordant molecular phylogenies support co-evolution of both eutherian-specific genes

**DOI:** 10.1186/s12862-015-0506-y

**Published:** 2015-10-12

**Authors:** Laurent Bianchetti, Yara Tarabay, Odile Lecompte, Roland Stote, Olivier Poch, Annick Dejaegere, Stéphane Viville

**Affiliations:** Biocomputing and Molecular Modelling Laboratory, Integrated Structural Biology Department, Genetics institute of Molecular and Cellular Biology (IGBMC), INSERM U964/CNRS UMR 1704/Strasbourg University, 1 rue Laurent Fries, 67404 Illkirch, France; Primordial Germ Cells’ Ontogeny and Pluripotency Laboratory, Functional Genomics and Cancer Department, Genetics Institute of Molecular and Cellular Biology (IGBMC), INSERM U964/CNRS UMR 1704/Université de Strasbourg, 1 rue Laurent Fries, 67404 Illkirch, France; Bioinformatics and Integrated Genomics Laboratory (LBGI), ICube, CNRS UMR 7357/Université de Strasbourg, 11 rue Humann, 67085 Strasbourg, France; Centre Hospitalier Universitaire, 67000 Strasbourg, France; Present address: Institut de génétique humaine (IGH), 141 rue de la Cardonille, 34396 Montpellier, France

**Keywords:** Co-evolution, Tex19, Sectm1, Molecular phylogeny, Expression, CXXC, Mammals, Transposon, Immunity

## Abstract

**Background:**

Transposable elements (TE) have attracted much attention since they shape the genome and contribute to species evolution. Organisms have evolved mechanisms to control TE activity. Testis expressed 19 (Tex19) represses TE expression in mouse testis and placenta. In the human and mouse genomes, Tex19 and Secreted and transmembrane 1 (Sectm1) are neighbors but are not homologs. Sectm1 is involved in immunity and its molecular phylogeny is unknown.

**Methods:**

Using multiple alignments of complete protein sequences (MACS), we inferred Tex19 and Sectm1 molecular phylogenies. Protein conserved regions were identified and folds were predicted. Finally, expression patterns were studied across tissues and species using RNA-seq public data and RT-PCR.

**Results:**

We present 2 high quality alignments of 58 Tex19 and 58 Sectm1 protein sequences from 48 organisms. First, both genes are eutherian-specific, *i.e.,* exclusively present in mammals except monotremes (platypus) and marsupials. Second, Tex19 and Sectm1 have both duplicated in *Sciurognathi* and *Bovidae* while they have remained as single copy genes in all further placental mammals. Phylogenetic concordance between both genes was significant (*p*-value < 0.05) and supported co-evolution and functional relationship. At the protein level, Tex19 exhibits 3 conserved regions and 4 invariant cysteines. In particular, a CXXC motif is present in the N-terminal conserved region. Sectm1 exhibits 2 invariant cysteines and an Ig-like domain. Strikingly, Tex19 C-terminal conserved region was lost in *Haplorrhini* primates while a Sectm1 C-terminal extra domain was acquired. Finally, we have determined that Tex19 and Sectm1 expression levels anti-correlate across the testis of several primates (ρ = −0.72) which supports anti-regulation.

**Conclusions:**

Tex19 and Sectm1 co-evolution and anti-regulated expressions support a strong functional relationship between both genes. Since Tex19 operates a control on TE and Sectm1 plays a role in immunity, Tex19 might suppress an immune response directed against cells that show TE activity in eutherian reproductive tissues.

**Electronic supplementary material:**

The online version of this article (doi:10.1186/s12862-015-0506-y) contains supplementary material, which is available to authorized users.

## Background

Genome stability is required to pass genetic information through generations. However, genetic variations are also crucial to generate organisms that could survive environmental changes. Transposable elements (TE) play a major role in genome evolution [1]. TE act as sites of recombination and can modify the expression of neighbor genes. Among TE, retrotransposons use a RNA intermediate to replicate and integrate back into the genome. Retrotransposons are mainly divided in 3 groups [2]: long interspersed nuclear element (LINE), short interspersed nuclear element (SINE), and long terminal repeat (LTR) also referred to as endogenous retroviruses (ERV). LINE-1, *i.e.,* a sub-class of LINE, encodes two proteins, *i.e.,* ORF1 and ORF2, while ERV encodes *gag*, *pro*, *pol* and *env* proteins. Organisms have evolved mechanisms to control retrotransposon activity [3], *e.g.,* transcriptional silencing by DNA methylation. Recently, the Tex19 gene was proposed to operate a genome defense mechanism against MMERVK10C and LINE-1 retrotransposons in mouse testis and placenta, respectively [4–6]. However, Tex19 molecular mechanism to control TE activity is still unknown.

Tex19 is a mammalian-specific [7] and orphan gene, *i.e.,* the encoded protein does not share sequence similarity with any known proteins [8]. Tex19 shows 2 paralogs in mouse, Tex19.1 and Tex19.2, that code for proteins of 351 and 317 residues, respectively. Initially, human was thought to exhibit no Tex19 ortholog [9, 10]. However, a human Tex19 gene was detected in GRCh37/hg19 genome assembly and was predicted to code for a 164 residue protein [7]. Human Tex19 protein is significantly shorter than mouse orthologs because a stop codon prematurely disrupts the coding sequence (CDS). Human Tex19 and mouse Tex19.1 protein sequences share 47 % identity and 56.8 % similarity. In mouse, Tex19.1 and Tex19.2 paralogs share 61 % identity and 69 % similarity and are specifically expressed in reproductive tissues [11]. Briefly, both Tex19.1 and Tex19.2 are expressed in testis while only Tex19.1 is expressed in placenta. Tex19.1 deletion leads to spermatogenesis defects and reduces perinatal survival [6]. Moreover, surviving adult Tex19.1 −/− male mice exhibit activation of MMERVK10C endogenous retroviruses and genomic DNA double strand breaks during meiosis [4, 6]. Finally, immunohistochemistry experiments localized Tex19.1 protein in the cytoplasm. Tex19.2 role is unknown. In the human and mouse genomes, Tex19 and Sectm1 are neighbors on chromosome cytoband 17q25.3 and 11qE2, respectively, but are not homologs.

A Sectm1 cDNA was originally cloned from human K562 erythroleukemic cells and codes for a 248 amino-acid protein which exists as a Golgi localized type I transmembrane (TM) protein and a secreted isoform [12]. Mouse exhibits 2 Sectm1 paralogs, *i.e.,* Sectm1a and Sectm1b, that are both expressed in a large variety of unrelated tissues [13, 14], *e.g.,* colon, kidney, stomach, testis, placenta, lung, thymus, spleen and liver. So far, Sectm1 has exclusively been studied in human and mouse and its phylogeny is unknown. Interestingly, Sectm1 binds CD7 [13], an Ig domain protein which is encoded by Sectm1 neighbor gene in the human and mouse genomes. In both organisms, Tex19, Sectm1 and CD7 are located in syntenic chromosome regions. In mouse, gene knock-out (KO) studies of Sectm1 or CD7 have not been carried out. CD7 is expressed on T, NK and pre-B lymphocytes. Sectm1 and CD7 protein complex modulates T-cell activation. In addition, Sectm1 expression is stimulated by interferon gamma (IFN-gamma) [15]. Sectm1 is thus clearly involved in the immune system.

Comparative genomics holds tremendous promises to fill the gap between sequence, structure, function and evolution. With the multiplication of 2^nd^ and 3^d^ generation sequencers [16, 17], large genomes can now be sequenced at an unprecedented pace and more than 50 mammalian genome projects are under way [18]. Mammals form a complex group of approximately 5,300 species divided in *Prototheria* (monotremes), *Metatheria* (marsupials) and *Eutheria* (usually called placental mammals). *Eutheria* are further divided in 4 groups, *i.e., Xenarthra* (*e.g.,* armadillo and sloth), *Afrotheria* (*e.g.,* elephant and rock hyrax), *Laurasiatheria* (*e.g.,* horse and shrew) and *Euarchontoglires* (*e.g.,* human and mouse) [19]. Species have been thoroughly selected for whole genome sequencing to achieve divergence across mammals and enable biologists to carry out unbiased molecular phylogenies. Classically, biologists wish to study gene evolutionary behavior by analyzing presence/absence, copy number, evolutionary rates, and genetic loci reshuffling across genomes. Such research projects may also benefit from expression data to provide support to biological conclusions. Complete genomes are particularly valuable because they provide quite definitive answers to gene presence/absence studies [20]. However, “finished” genome sequences may still contain some gaps. So far, only a few mammalian genomes have been completed, namely *Ornithorhynchus anatinus* (platypus) (*Prototheria*) [21], *Monodelphis domestica* and *Macropus eugenii* (*Methateria*) [22], and human and mouse (*Eutheria*). In a genome project, sequence annotation is a crucial step. Bioinformatics pipelines annotate sequences on a genome wide scale and pave the way to gene evolution studies. In particular, multiple alignments of complete protein sequences (MACS) [23] have proved invaluable to investigate sequence, structure, function and evolution relationships for countless proteins. Recently, specialized bioinformatics tools have made it possible to detect co-evolving genes [24]. Co-evolving proteins are comparative genomics niceties. Such proteins are characterized by concordant molecular phylogenies, *i.e.,* shared history of gene duplication and gene losses, and similar patterns of presence and absence across species [25]. In addition, co-evolution tends to indicate functional relationship, physical interaction or same biological process participation. Co-evolution has been reported for hormones and receptors, *e.g.,* insulin [26] and vasopressin [27], protease and substrate, *e.g.,* hatching enzyme and egg envelope protein [28]. *Cis* proximity of co-evolving genes along the chromosome is not required, *e.g.,* the insulin and its receptor are coded by genes located within human chromosome 11 and 19 [26], respectively. In prokaryotes, gene order along the genome and function are related and genes are organized in operons [29]. By contrast, operons are very rare in eukaryotes [30, 31]. Only a few gene systems, *e.g.,* Hox, β-globin, GPAT/AIRC [32] and invertebrate species [33] exceptions have been reported. As a result, *cis* proximity of genes along eukaryote chromosomes does not relate to function and molecular phylogeny.

High-throughput (HTP) transcript sequencing across tissues and species now enables biologists to study gene expression through the lens of evolution [34, 35]. However, molecular phylogenetic analysis is recommended before profiling gene expression [36, 37]. Gene expression patterns across tissues and species may provide valuable information to elucidate gene function. Among expression profiling methods, RNA-seq has been a breakthrough to quantify gene expressions on a genome wide scale. A plethora of RNA-seq experiments carried out in mammalian tissues has been made publicly available in the sequence read archive (SRA) database [38].

Using a 13 protein sequence alignment, we previously established the mammalian specificity of Tex19 [7]. The recent availability of a series of mammalian genome sequences for *Prototheria*, *Metatheria* and *Eutheria* prompted us to revisit Tex19 molecular phylogeny. Intriguingly, we noticed that both Tex19 and Sectm1 were unique in human but duplicated in mouse and rat, *i.e.,* a quite unusual gene copy number pattern. In addition, both genes were neighbors on human, mouse and rat genomes. This unusual gene system and loci proximity in human and rodents encouraged us to simultaneously investigate Tex19 and Sectm1 molecular phylogenies. In the present study, 2 sequence homology searches have been carried out in protein databases and 2 high quality MACS of 58 proteins have been built. First, the sequence homologs searches show that both genes are eutherian-specific. Second, Tex19 and Sectm1 have duplicated in *Sciurognathi* and *Bovidae* while they have been maintained as single copy genes in all further placental mammals. The remarkable concordance of the evolutionary histories of the two genes, and in particular the duplications in the same subset of species strongly suggests co-evolution and functional relationship. Finally, Tex19 and Sectm1 expressions were analyzed across tissues and species and enabled us to detect pattern oppositions, *e.g.,* human placenta expresses Sectm1 but not Tex19 while it is exactly the opposite in mouse placenta. Since Tex19 represses TE expression in reproductive tissues and Sectm1 plays a role in immunity, our results suggest a new eutherian-specific mechanism that might prevent the immune system from attacking tissues where controlled TE activity occurs.

## Methods

### MACS construction

We used PipeAlign [39] to generate the MACS. Homologs of mouse full-length Tex19.1 and human full-length Sectm1 proteins were searched in Uniprot, RefSeq and the Protein Data Bank (PDB) using BLASTp (expect threshold 10^−4^, word size 3, BLOSUM62, gop 11 and gep 1). For Tex19 and Sectm1 homolog searches, the score, identities and positives cut-off values were set to 65 bits, 28 % and 40 %, respectively. In addition, whole genome shotguns (WGS) were searched by tBLASTn for further homologs. The mouse Tex19.1 sequence was used as a query because human Tex19 lacks the C-terminal region. Conversely, the human Sectm1 sequence was used as a query because mouse Sectm1 proteins lack the C-terminal region. Alignment quality was manually refined in the SeqLab (GCG, Wisconsin package) editor by moving residues. For each protein, basic data, *i.e.,* accession number, organism, brief taxonomy, sequence length and coding origin were reported in a file (publicly available on the FigShare repository http://figshare.com/articles/Catalog_of_Tex19_protein_sequences/1491365 and http://figshare.com/articles/Catalog_of_Sectm1_protein_sequences/1491366). Multiple alignment schemas were drawn in Jalview 2 [40]. Sequence logos were created in WebLogo 2.8.2 [41].

### Sequence comparisons and secondary structure predictions

Pairwise sequence comparisons were carried out with Needleman & Wunsch or Smith & Waterman algorithms implemented in the needle and water EMBOSS tools (Olson SA) (default parameters), respectively. Secondary structures were predicted with PSIPRED (Jones DT). Position-specific-iterated BLAST (PSI-BLAST) were carried out in the NCBI nr protein database with default parameters to search for remote homologs of Tex19 and Sectm1. Ten and three iterations were carried out for Tex19 and Sectm1, respectively.

### Molecular modelling

A HHpred search was carried out in the PDB with human Sectm1 as a query. The [PDB:3SOB] structure (X-ray, 1.9 Angström resolution) was selected as a template to model by homology Sectm1 Ig-like domain in the Modeller 9.13 program (Sali A). Sectm1 3D model was validated using MolProbity (Chen V. B. et al., 2010) and PROSA (Wiederstein M. and Sippl M. J., 2007). 3D structures were analyzed in PyMOL 1.4.1 (Schrödinger, LLC.).

### Molecular phylogeny

The maximum likelihood (ML) algorithm implemented in MEGA6 [42] was applied to build the phylograms of all full-length sequences. Using ProtTest [43], the Jones-Taylor-Thornton (JTT) residue substitution model was selected as the most appropriate. A 500 replicate bootstrap was carried out. Finally, phylograms were annotated with the iTOL software [44].

### RNA-seq profiles across tissues and species

RNA-seq experiments were selected from the SRA database. Tex19 and Sectm1 transcripts were masked for interspersed repetitive elements (IRE) (RepeatMasker, Smit A, unpublished) and used as queries on selected RNA-seq experiments. Reads were mapped to Tex19 and Sectm1 masked transcripts using MegaBLAST. Thanks to its default large word size (W = 28), MegaBLAST selectively retrieves sequences that are highly similar to a query. In double paralog species, *i.e.,* mouse, rat and cow, a best score approach was applied to reliably map reads to their proper paralog. E*x-aequo* scoring reads were discarded. *Homo sapiens* [RefSeq:NM_207459], *Pan troglodytes* [RefSeq:NM_001246469], *Pan paniscus* [RefSeq:XM_003808957], *Papio anubis* [RefSeq:XM_003913613] and *Macaca mulatta* [RefSeq:NM_001194590] Tex19 transcripts were used as queries. Sectm1 expression was searched with *Homo sapiens* [RefSeq:NM_003004], *Pan troglodytes* [RefSeq:XM_001167936], *Papio anubis* [RefSeq:XM_003913612] and *Macaca mulatta* [Genbank:EHH25327] transcript sequences as queries. Mouse experiments were searched with Tex19.1 [RefSeq:NM_028602], Tex19.2 [RefSeq:NM_027622], Sectm1a [RefSeq:NM_145373] and Sectm1b [RefSeq:NM_026907]. Finally, cow Tex19.alpha [RefSeq:XM_002696130], Tex19.beta [RefSeq:XM_001251766], Sectm1.alpha [RefSeq:NM_001102326] and Sectm1.beta [RefSeq:NM_001083787] were used as queries on cow RNA-seq experiments.

### EST profiles

Human and cow placenta ESTs were automatically selected from Genbank release 199 using taxonomy and tissue_type fields. Genbank records were converted to BLAST databases. Human Sectm1 [RefSeq:NM_003004] and cow Tex19.alpha [RefSeq:XM_002696130], Tex19.beta [RefSeq:XM_001251766], Sectm1.alpha [RefSeq:NM_001102326] and Sectm1.beta [RefSeq:NM_001102326] masked transcripts were used as queries. MegaBLAST was used to search the EST database. For cow, ESTs were properly mapped to Tex19 or Sectm1 paralogs using a best score approach. E*x-aequo* scoring ESTs were discarded.

### Reverse-transcription polymerase chain reaction (RT-PCR)

RNA from selected tissues was prepared using the MACHEREY-NAGEL protocol of the NucleoSpin RNA L kit following the manufacturer’s instructions. After DNase I digestion (Roche), 2 μg RNA was reverse-transcribed by random priming using Superscript II (Invitrogen). Resulting cDNA was diluted in a final volume of 80 ml. 1 μL of each cDNA was used to perform 40 cycles PCR amplification of 94 °C for 30s, 64 °C for 1 min 30s and 72 °C for 1 min 30s. PCR products were checked by sequencing (GATC) to validate the specificity of the primers. In rat, 5′-ATGTGTCCCCCAGTCAGTGTT-3′ (forward) and 5′-TCAAGGGAAGAAGGATCGAGCA-3′ (reverse) primers were used to target Tex19.1 (NM_001017482) while 5′-ATGTGTCCCCCAGTCAGTGTT-3′ (forward) and 5′-TTAGTTGTGTGGCTCAGGGGA-3′ (reverse) targeted Tex19.2 (NM_001109622). In cow, 5′-CGATTCTGAGGCATGGTCTG-3′ (forward) and 5′ GTATGATTGTGCAAGCCCAC 3′ (reverse) primers were designed to target the XM_002696130 transcript sequence, which codes for XP_002696176, *i.e., Bos taurus* Tex19.alpha. 5′-AACTCAGAGACAGGGTCTGA-3′ (forward) and 5′-AAGTGATCATCTAGGTCCAT-3′ (reverse) primers targeted the XM_005195806 transcript which codes for XP_005195863, *i.e., Bos taurus* Tex19.beta. 5′-ATGGTGAAGGTCGGAGTGAA-3′ (forward) and 5′-TTACTCCTTGGAGGCCATGT-3′ (reverse) primers were used to target the cow GAPDH transcript.

## Results

### Tex19 and Sectm1 are not homologs

A dot plot was drawn to determine if Tex19 and Sectm1 proteins exhibit sequence similarity (Additional file 1). As controls of sequence homology, human Tex19 and Sectm1 were compared to mouse Tex19.1 and Sectm1a orthologs, respectively. Human Tex19 and Sectm1 proteins do not show any similarity while homology between human and mouse orthologs was obvious. To determine if Tex19 and Sectm1 proteins might be distant homologs, *i.e.,* structure was conserved while sequences diverged, folds were investigated. First, Sectm1 structure is unknown. However, a weak sequence similarity with the Ig domain was previously mentioned [12]. Second, Tex19 structure has not been resolved yet. Using Tex19 protein sequence as a query, a conserved domain database (CDD) search did not produce any hit. Therefore, Tex19 unlikely folds into an Ig domain. In conclusion, both proteins do not share sequence homology and are unlikely distant homologs.

### Tex19 and Sectm1 neighbor genes are unique in human and duplicated in mouse and rat

**Fig. 1:**
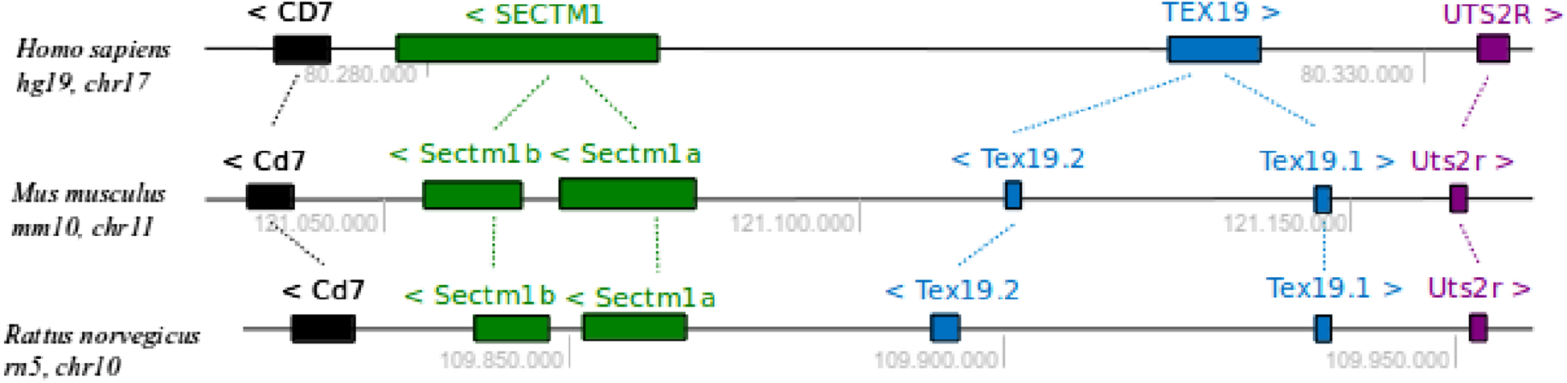
Schematic syntenic map of Tex19 and Sectm1 genomic loci across human, mouse and rat *Homo sapiens* GRCh37/hg19, *Mus musculus* GRCm38/mm10 and *Rattus norvegicus* RGSC5.0/rn5 genome assemblies were used. Black, green, blue and magenta boxes represent CD7, Sectm1, Tex19 and Uts2r genes, respectively. Thin dashed lines join orthologs. “>” and “<” symbols indicate gene transcription orientations. Light gray numbers show chromosome position0073

In human, the presence of a Tex19 ortholog has been controversial [7, 9]. Therefore, we wished to determine the exact number of homologs on the latest human genome assembly. In our hands, exhaustive sequence similarity searches on the reference GRCh37.p13/hg19 genome showed that *tex19,* but also *sectm1* are unique. Moreover *tex19* and *sectm1* are neighbors (Fig. 1) and localize on cytoband 17q25.3 on the human genome. A Tex19.1 and Tex19.2 paralogy was previously reported in mouse and rat [7]. Tex19.1 and Tex19.2 genes both localize on mouse chromosome cytoband 11qE2. To determine if the mouse genome codes for further Tex19 homologs, an exhaustive tBLASTn search with Tex19.1 as a query was carried out. Two significant hits were produced, both on chromosome 11. Therefore, Tex19 shows 2 paralogs in mouse. An exhaustive search on the mouse genome with Sectm1a as a query produced 2 significant hits, both on chromosome 11. As a result, mouse exhibits also 2 Sectm1 paralogs. In rat, Tex19.1 and Tex19.2 genes are both localized on chromosome cytoband 10q32.3. Using the same approach, we determined the presence of 2 Tex19 and 2 Sectm1 genes in rat. Conclusively, Tex19 and Sectm1 are unique in human while duplicated on mouse and rat genomes. We compared Tex19 and Sectm1 genomic loci across species. Tex19 and Sectm1 are neighbors on human, mouse and rat chromosome cytoband 17q25.3, 11qE2 and 10q32.3, respectively. Since the rodents and human radiation, *i.e.,* 90 million years ago, Tex19 and Sectm1 gene copies have been evolutionarily kept close to each other on the genome. In human, *tex19* and *sectm1* are separated by a 25 kb intergenic region and both genes are transcribed in opposite directions. In mouse and rat, Tex19.2 and Sectm1a are separated by 35 and 31 kb DNA regions, respectively. In the 3 species, CD7 is 5' to Sectm1 while the Urotensin 2 receptor (Uts2r) is 3' to Tex19. The chromosome region between CD7 and Uts2r spans approximately 63, 126, and 149 kb in human, mouse and rat, respectively. While no functional relation has been reported between Tex19 and Uts2r, Sectm1 protein binds CD7. Sectm1 and CD7 are thus clustered genes on the genome. In both rodents, Sectm1a and Sectm1b are transcribed in the same direction while Tex19.1 and Tex19.2 show opposite transcription directions.

### Tex19 and Sectm1 are both eutherian-specific

Using sequence homology searches across genomes and an alignment of 13 Tex19 proteins originating from 10 species, we previously reported Tex19 mammalian specificity [7]. Now, we present an extended alignment of 58 proteins from 48 organisms supporting Tex19 eutherian specificity (available at http://figshare.com/articles/Tex19_multiple_alignment_of_complete_protein_sequences/1491363). Exhaustive tBLASTn searches across prokaryote, plant, yeast, invertebrate, fish, and bird genomes retrieved no Tex19 homologs (Table 1). Moreover, *Ornithorhynchus anatinus* (platypus), *Macropus eugenii* (wallaby) and *Monodelphis domestica* (opossum) complete genomes did not produce any significant hit. Therefore, Tex19 is absent in monotremes and marsupials. Tex19 was further searched in the genomes of organisms that represent the 4 super-orders of eutherians (Additional file 2), *i.e., Afrotheria, Laurasiatheria, Euarchontoglires* and *Xenarthra.* Without ambiguity, Tex19 homologs were detected in *Afrotheria* (3 sequences from 3 species), *Laurasiatheria* (25 sequences from 20 species) and *Euarchontoglires* (30 sequences from 25 species). No Tex19 homolog was detected in *Xenarthra*. Since Xenarthra genomes, *e.g., Dasypus novemcinctus* (armadillo) and *Choloepus hoffmanni* (sloth) are not finished yet, we cannot conclude that Tex19 does not exist in those species. Thus, except in Xenarthra where Tex19 presence/absence is ambiguous, Tex19 appears to be eutherian-specific. A pairwise sequence comparison of human and mouse Sectm1 proteins was previously published [14]. We present a Sectm1 MACS of 58 proteins from 48 organisms (available at http://figshare.com/articles/Sectm1_multiple_alignment_of_complete_protein_sequences/1491364). First, Sectm1 is absent in non-mammalian species, monotremes and marsupials (Table 1). Second, Sectm1 homologs have been detected in *Afrotheria* (5 sequences from 5 species), *Laurasiatheria* (28 sequences from 23 species), *Euarchontoglires* (24 sequences from 19 species) and *Xenarthra* (1 sequence from 1 species). Indeed, the *Dasypus novemcinctus* [RefSeq:XP_004478808] protein shares significant sequence similarity with the human Sectm1 (45.7 % identity and 56.3 % similarity). Therefore, we can conclude that Sectm1 is present in the genome of organisms that represent the 4 super-orders of eutherians. Thus, Sectm1 also appears to be eutherian-specific.

**Table 1:** Tex19 and Sectm1 presence/absence across species

Eutherian specificity of Tex19 and Sectm1				
Tex19	Sectm1	Organism	Clade	Eutheria
A	A	*Escherichia coli* ^a^	Bacteria	no
A	A	*Saccharomyces cerevisiae* ^a^	Fungi	no
A	A	*Arabidopsis thaliana* ^a^	Plant	no
A	A	*Caenhorhabditis elegans* ^a^ *, Drosophila melanogaster* ^a^	Invertebrate	no
A	A	*Brachydanio rerio* ^a^	Fish	no
A	A	*Gallus gallus*	Bird	no
A	A	*Ornithorhynchus anatinus* ^a^	Monotreme	no
A	A	*Macropus eugenii* ^a^ *, Monodelphis domestica* ^a^	Marsupial	no
A	P	*Dasypus novemcinctus* ^b^	Xenarthra	yes
P	P	*Bos taurus*	Bovidae	yes
P	P	*Cavia porcellus*	Hystricognathi	yes
P	P	*Mus musculus* ^a^ *, Rattus norvegicus*	Sciurognathi	yes
P	P	*Homo sapiens* ^a^ *, Pan troglodytes* ^a^ *, Macaca mulatta*	Primate	yes

*A* absent, *P* present, ^(a)^complete genome, ^(b)^requires finished genome confirmation

**Table 2:** Tex19 and Sectm1 gene copies across species

Gene copies of Tex19 and Sectm1 across Eutheria			
Tex19	Sectm1	Eutheria	Clade
0	1	*Dasypus novemcinctus* ^b^	Xenarthra
1	1	*Homo sapiens* ^a^	Primate
1	1	*Pan troglodytes* ^a^	Primate
1	1	*Macaca mulatta*	Primate
1	1	*Cavia porcellus*	Hystricognathi
2	2	*Mus musculus* ^a^	Sciurognathi
2	2	*Rattus norvegicus*	Sciurognathi
2	2	*Bos taurus*	Bovidae

^(a)^complete genome, ^(b)^requires finished genome confirmation

### Remarkable concordance of Tex19 and Sectm1 molecular phylogenies

**Fig. 2:**
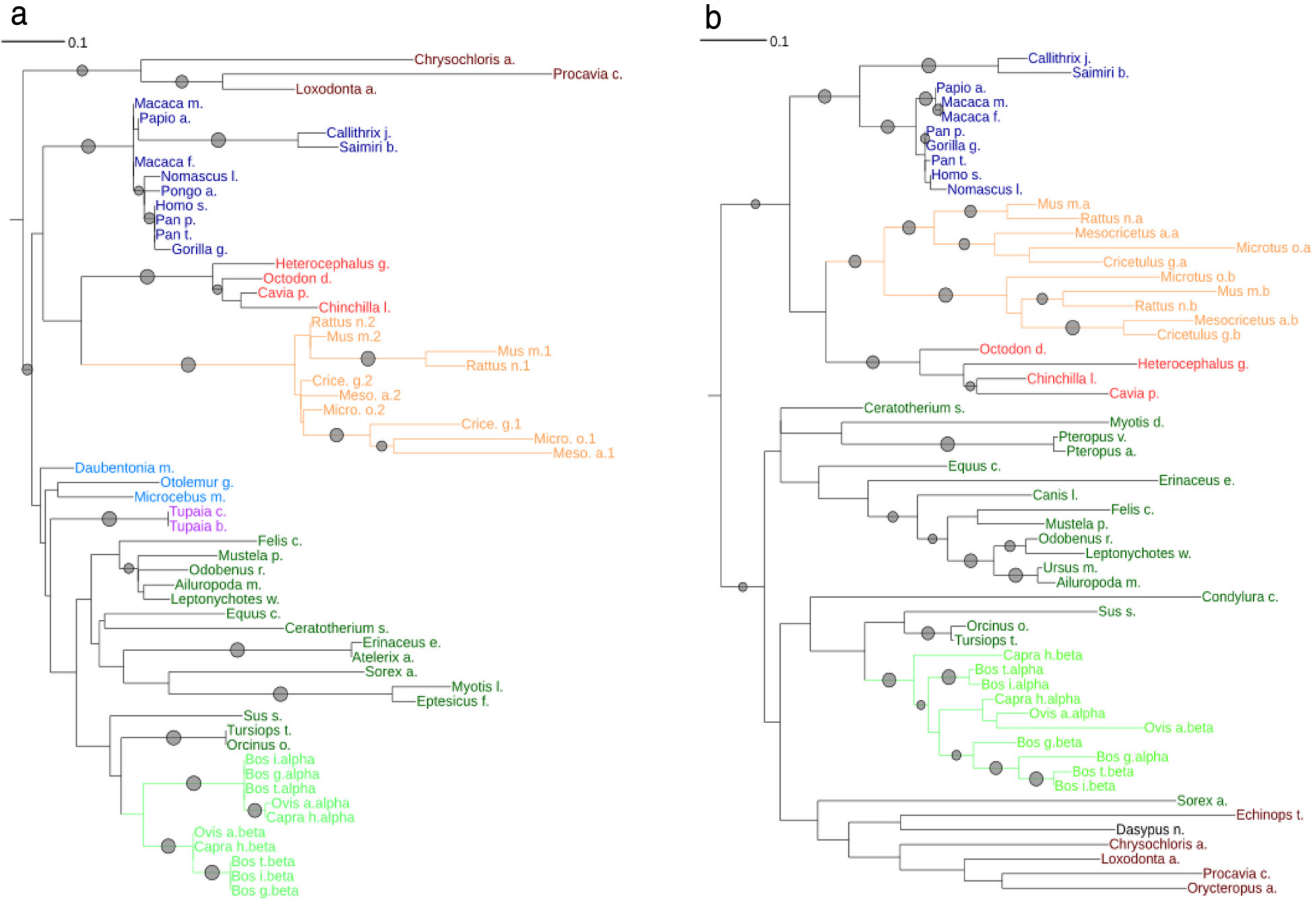
Tex19 and Sectm1 ML phylograms of all eutherian sequences. **a** Tex19 and **b** Sectm1. Bootstrap values greater than 65 % are symbolized by gray circles. Taxonomic groups of species are colored as follows: *Haplorrhini* = dark blue, *Strepsirrhini* = light blue, *Scandentia* = purple, *Afrotheria* = dark brown, *Hystricognathi* = dark red, *Sciurognathi* = orange, *Bovidae* = light green, further *Laurasiatheria* = dark green. Orange and light green branches depict *Sciurognathi* and *Bovidae* lineage sequences, respectively. Sectm1 sequences of *Strepsirrhini* species were not available in protein databases likely because genomes were still incomplete

Tex19 and Sectm1 concordant gene copy numbers in human, mouse and rat prompted us to infer the molecular phylogenies of both genes across species. First both genes are eutherian-specific. Second, both genes duplicated in *Sciurognathi* (a rodent suborder) and *Bovidae* (a family of *Ruminantia*) (Fig. 2). Third, in all further *Eutheria*, Tex19 (Fig. 2a) and Sectm1 (Fig. 2b) were both present as single-copy genes. Of note, *Sciurognathi* and *Bovidae* orthologs do not cluster together in the phylograms which supports 2 independent gene duplication events. We used 3 methods to test statistically the concordance of Tex19 and Sectm1 phylogenetic histories across *Eutheria*. First, we applied an independence Pearson’s Chi-squared test. Tex19 and Sectm1 gene copies were counted across 8 selected eutherian species from *Xenarthra* to human (Table 2) and a contingency table was drawn (Table 3). A 0.018 *p*-value was calculated and was below 5 % type I error risk. Therefore, we concluded that Tex19 and Sectm1 gene copy numbers were not independent across *Eutheria* which supports co-evolution. Second, identity percent vectors to a selected reference sequence, *i.e.,* human, were used to calculate the correlation between both protein sequences across *Eutheria* [25]. Pairwise identity percents were calculated for each protein in the MACS. Two identity percent vectors were generated, 1 for Tex19 and 1 for Sectm1, and correlated at 0.78 (Pearson) providing further support to Tex19 and Sectm1 co-evolution. Third, the MirrorTree server (Ochoa D. and Pazos F., 2010) was used to calculate the similarity between both trees (all sequences). 39 species were common to both phylograms, a 0.717 correlation and a *p*-value < = 10^−6^ were calculated and supported co-evolution.

**Table 3:** Eutheria contingency table by Tex19 and Sectm1 gene copy pattern

	Tex19: 0 copy	Tex19: 1 copy	Tex19: 2 copies
Sectm1: 1 copy	1	4	0
Sectm1: 2 copies	0	0	3

### Tex19 shows 4 invariant cysteines and *Haplorrhini* lost a conserved C-terminal region

**Fig. 3:**
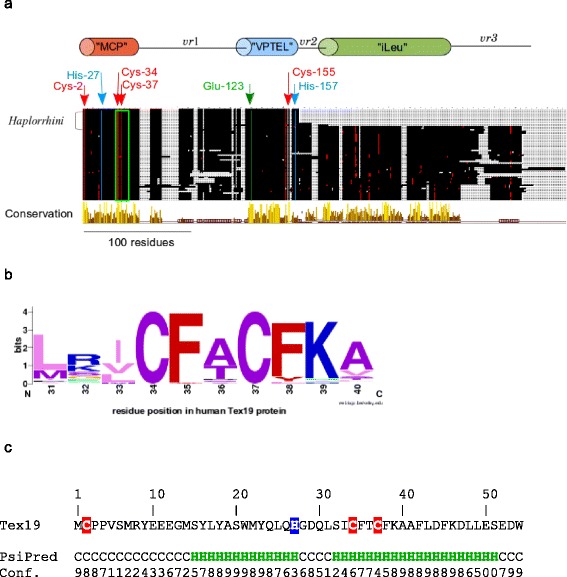
Tex19 protein organization. **a** MACS schema. Colored cylinders represent conserved regions while “*vr*” stand for variable regions. Cysteines are depicted as red dots while any other residues, except invariant amino-acids, are shown in black. Gray surfaces represent gaps. Arrows indicate invariant residues and offsets refer to the human sequence, *i.e.,* [RefSeq:NP_997342]. The open green rectangle shows the position of the C_34_F[AT]C_37_[FY] motif. *Haplorrhini* sequences were clustered at the top of the alignment. **b** C_34_F[AT]C_37_[FY] logo. **c** Secondary structure prediction in conserved “MCP” region (PsiPred), *i.e.,* from residue Met-1 to Trp-54. H and C symbols represent alpha-helix and coil predicted residues, respectively. Confidence value of secondary structure prediction is reported for each residue (maximum confidence equals 9). Invariant cysteines are depicted with a red background while invariant His-27 is shown with a blue background

Basing our analysis on a 13 sequence alignment, we previously reported the presence of 3 conserved regions in Tex19 that we named “MCP”, “VPTEL” and “non-primate specific” from the N to the C-terminus. Now, the 58 Tex19 MACS enables us to confirm those 3 conserved regions and the invariance of the Cys-2 and Glu-123 (offsets refer to human Tex19) that are involved in the “MCP” and “VPTEL” motifs (Fig. 3a), respectively. The “non-primate specific” region is conserved in all eutherian species including *Strepsirrhini*, *e.g., Otolemur garnettii,* except in *Haplorrhini* primates that remarkably lost this region (Fig. 3a). Since *Strepsirrhini* are primates, the C-terminal region cannot be further called “non-primate specific” and shall be renamed. Remarkably, the 58 Tex19 MACS reveals 4 invariant cysteines. In the human sequence, 3 invariant cysteines are localized at position 2, 34 and 37, *i.e.,* in the “MCP” region, and the latter at position 155, *i.e.,* in the “VPTEL” region. Cys-34 and Cys-37 are involved in a C_34_F[AT]C_37_[FY] motif (Fig. 3b) which fits the fuzzier CXXC pattern. The “MCP” region contains also a highly conserved histidine at position 27. Moreover, a further invariant residue, *i.e.,* His-157, is present in the “VPTEL” region. In the previously called “non-primate specific” region, a series of leucine amino-acids are invariant at positions 186, 188, 240, 241, 270, 273, 282, and 289 (offsets refer to mouse Tex19.1 protein). Therefore, we propose to rename the “non-primate specific” region as “iLeu” for invariant leucines. Tex19 does not show any sequence similarity with known proteins or domains. Strategies have been described to detect remote homology [45], *i.e.,* fold conservation between proteins that share weak primary sequence similarity. Using the human “MCP” region sequence (Met-1 to Trp-54) as a query, remote homolog search algorithms, *e.g.,* PSI-BLAST, Interproscan and HMMER, did not produce any significant hit. Human Tex19 “MCP” region was submitted to PSIPRED secondary structure prediction program which predicted 2 alpha-helices (Fig. 3c). The first helix spans residues Ser-15 to His-27 with a 7.4 +/− 1.8 confidence value while the second helix spans residues Ser-32 to Ser-51 with a 6.05 +/− 2.8 confidence value (maximum confidence is 9). Invariant Cys-2 and His-27 amino-acids were predicted in a coil region and in the first alpha-helix, respectively. Invariant Cys-34 and Cys-37, *i.e.,* the C_34_F[AT]C_37_[FY] motif, were both predicted in the second alpha-helix.

### Sectm1 exhibits an Ig-like fold with an atypical disulfide bridge and *Haplorrhini* acquired an extra C-terminal region

**Fig. 4:**
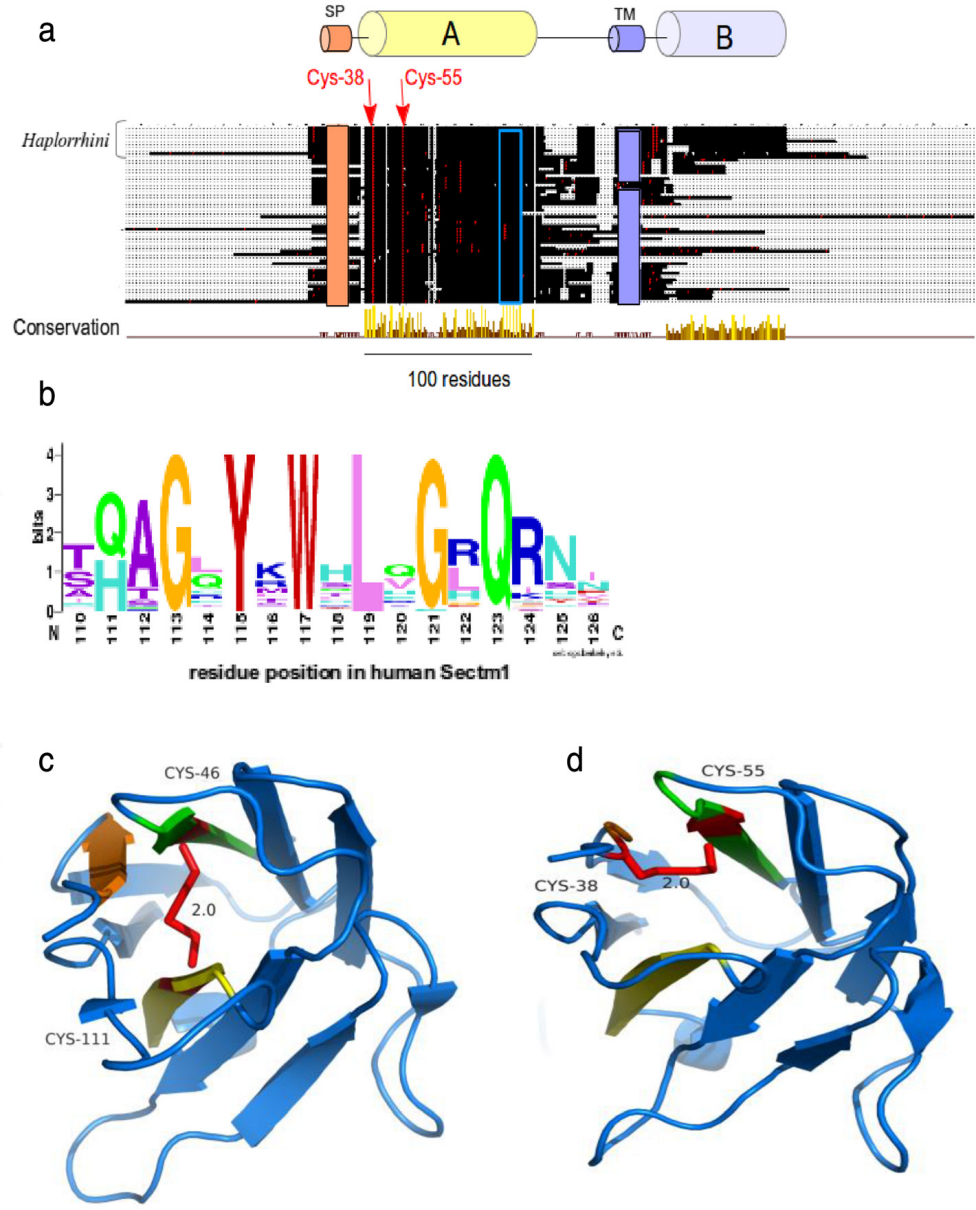
Sectm1 protein organization. **a** MACS schema. Colored cylinders represent conserved regions, *e.g.,* region A is the Ig-like domain, while thin lines stand for variable regions. Cysteines are depicted as red dots while any other residues are shown in black. The orange rectangle symbolizes a SP. The violet rectangle delineates a TM. The open blue rectangle shows the position of the G_113_XYXWXLXGXQ_123_ highly conserved motif. Arrows indicate 2 invariant cysteines. Amino-acid offsets refer to the human sequence, *i.e.,* [RefSeq:NP_002995]. *Haplorrhini* sequences were clustered at the top of the alignment. **b** Highly conserved G_113_XYXWXLXGXQ_123_ motif logo; (**c**) Classical Ig domain [PDB:3SOB] used for homology modelling showing a disulfide bridge (red) between beta-sheets. **d** 3D model of Sectm1 Ig-like domain, the atypical disulfide bond that is predicted to join both invariant cysteines is depicted in red

No multiple alignment of Sectm1 has been proposed so far. However, a secretion peptide (SP), a transmembrane (TM), and a weak sequence similarity with the Ig domain were previously mentioned [12]. Using the 58 Sectm1 MACS, we report the presence of a 100 residue conserved region, that we called “A” (Fig. 4a). Strikingly, *Haplorrhini* primates exhibit an extra C-terminal region that we named B. Conserved region A spans residue Trp-33 to Val-133 (residue offsets refer to the human sequence). Region A shows 2 invariant cysteines, *i.e.,* Cys-38 and Cys-55. In addition, a strongly conserved motif containing 6 invariant residues G_113_XY_115_XW_117_XL_119_XG_121_XQ_123_ (Fig. 4b) is present in region A. A sequence similarity between Sectm1 and the Ig domain was previously reported using BLASTp or FASTA tools [12]. Using PSI-BLAST and only 3 iterations, we were also able to reproduce this result and a significant sequence similarity was found between human Sectm1 conserved region A and the human B-cell antigen receptor complex-associated protein alpha chain (CD79A, [RefSeq:NP_067612]) (Table 4). A conserved domaine database (CDD) search with human CD79A sequence as a query retrieved the Ig domain without ambiguity (E-value = 3.85e-08). However, CD79A structure has not been resolved yet. Therefore, CD79A could not be used as a template for homology modelling. Using human Sectm1 sequence from residue 1 to 186 (*i.e.,* residues that aligned on CD79A) as a query, a HHpred search [46] in the structure database retrieved [PDB:3SOB_L] (Score 48.5, E-value = 0.0013), *i.e.,* an antibody light chain (Table 5). A predominant feature of most Ig domains is a sandwich of 2 beta-sheets connected by a disulfide bridge [47]. The classical disulfide bridge was present in [PDB:3SOB_L] template structure between Cys-46 and Cys-111 (Fig. 4c). Using 3SOB_L as a template, the 3D structure of Sectm1 Ig-like domain could be modelled (Fig. 4d). The model was submitted to ProSA [48] which calculated an overall quality score of −4.21, *i.e.,* in the range of native protein structures. Knowledge-based energy of the molecule was in an acceptable range. Moreover, using MolProbity, Phi and Psi dihedral angles of the Sectm1 Ig-like model were checked. 96 % (96/100) of all residues showed dihedral angles in Ramachandran plot favored regions. However, a peculiarity of the Sectm1 sequence is that the conserved cysteine are rather close in sequence (Cys-38 and Cys-55) and situated in N-terminal of the fold with respect to classical Ig-domains, and they were therefore not aligned with those of the template. To ensure that it is possible to conserve the Ig–domain topology while forming a disulfide bridge between the conserved cysteine, a restraint was applied to bridge both Sectm1 invariant cysteine to construct the 3D model. The resulting disulfide bridge did not connect both beta-sheets as the classical Ig disulfide bridge does but a beta-strand and a loop (Fig. 4d) of the same beta-sheet. Sectm1 Ig-like domain may thus form an atypical disulfide bridge while maintaining the Ig-fold. Further structural studies would however be needed to obtain a high resolution 3D structure of Sectm1.

**Table 4:**
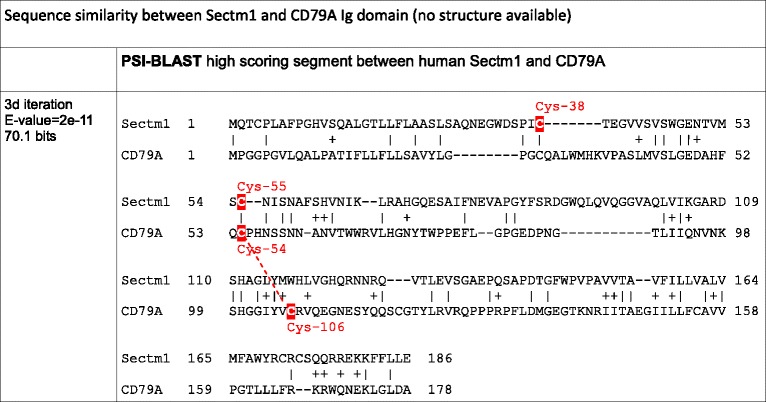
Template selection for homology modelling of Sectm1 Ig-like domain

Invariant cysteines are highlighted in red

CD79A putative disulfid bond is depicted as a red dashed line

**Table 5:**
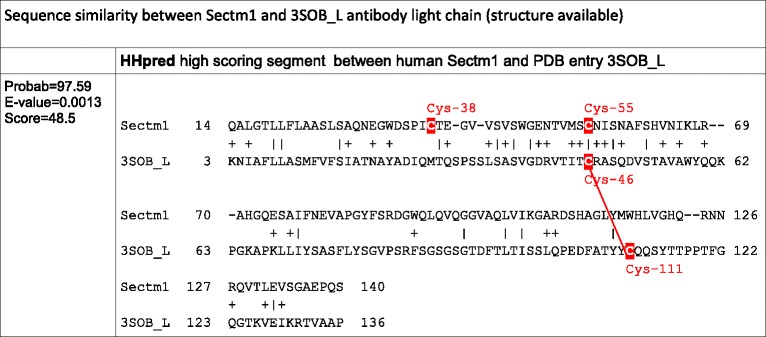
Template selection for homology modelling of Sectm1 Ig-like domain

Invariant cysteines are highlighted in red

3SOB disulfid bond is depicted as a red line

### Tex19 and Sectm1 expression patterns are opposite in testis and placenta across species

**Fig. 5:**
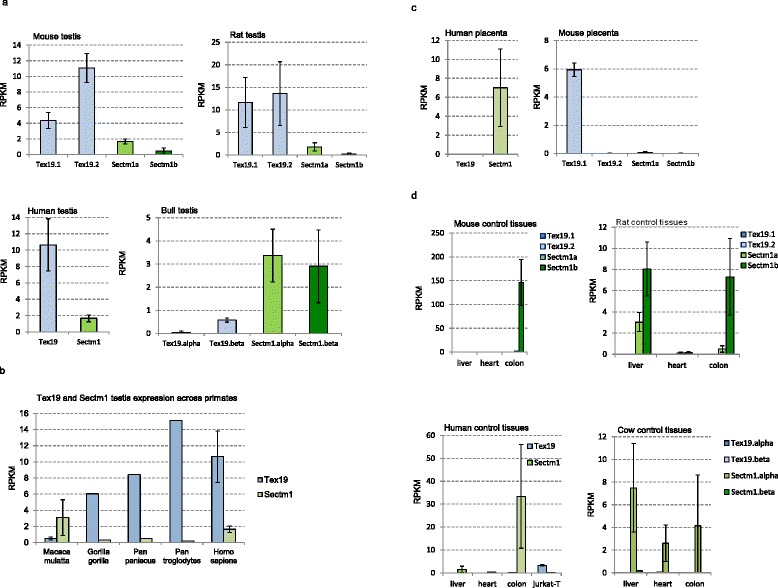
Tex19 and Sectm1 expression profiles across species and tissues using RNA-seq. Expression levels were normalized by RPKM. **a** Testis, human (*n* = 3), mouse (*n* = 4), rat (*n* = 3) and bull (*n* = 3). **b** Primate testis, human (*n* = 3), *Pan troglodytes* (*n* = 1), *Pan paniscus* (*n* = 1), *Gorilla gorilla* (*n* = 1), *Macaca mulatta* (*n* = 4). **c** Placenta, human (*n* = 6), mouse (*n* = 3). **d** Control tissues (*n* = 12)

**Fig. 6:**
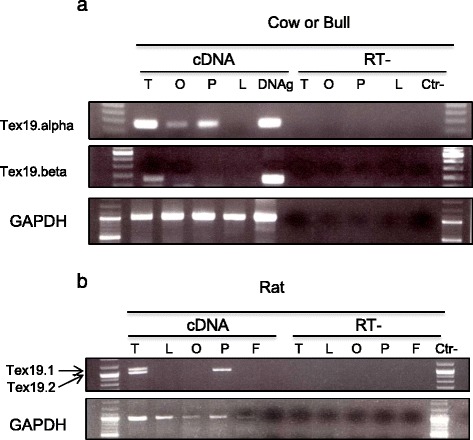
Reverse Transcription-PCR (RT-PCR). T: testis, O: ovary, P: placenta, L: liver, DNAg: genomic DNA, Ctr-: negative control, RT-: negative retrotranscribed samples. **a** Tex19.alpha and Tex19.beta RT-PCR in cow. **b** Tex19.1 and Tex19.2 RT-PCR in rat

Both gene expression profiles were investigated across species using publicly available RNA-seq data. In human, Sectm1 is expressed in a large variety of unrelated tissues, *e.g.,* colon, placenta, kidney, stomach, lung and thymus (Additional file 3). In mouse, it has been established that Tex19.1 and Tex19.2 are specifically expressed in reproduction and embryonic tissues, *i.e.,* testis, placenta, ovary and primordial germ cells (PGC) [11]. Therefore, we selected testis and placenta as reference tissues to simultaneously investigate both gene expressions in mouse, cow (or bull) and primates including human. 68 RNA-seq datasets, *i.e.,* 2.8 billion reads, were processed. Liver and heart served as Tex19 negative controls while colon was used as Sectm1 positive control. Moreover, Jurkat-T cells provided a human Tex19 positive control (data not shown). In cow, liver and heart were not strictly negative Sectm1 controls because both tissues expressed Sectm1. Because human, mouse, rat and cow are placental mammals, one may expect some similarity between orthologous gene expression profiles. Surprisingly, we observed heterogeneous or quite opposite patterns of Tex19 and Sectm1 expressions in testis and placenta across species. In testis, human, mouse and rat predominantly express Tex19 while bull mainly expresses Sectm1 (Fig. 5a). Strikingly, Tex19 and Sectm1 expression levels anti-correlate (−0.72 Pearson coefficient) across the testis of 5 selected primates (Fig. 5b) which supports anti-regulated gene expressions. In placenta, human expresses Sectm1 but not Tex19 while it is exactly the opposite in mouse (Fig. 5c). For cow, placenta RNA-seq experiments were not available. Therefore, expressed sequence tags (ESTs) were analyzed. EST detect only abundantly expressed genes. Both Sectm1.alpha (7 ESTs) and Sectm1.beta (42 ESTs) transcripts were retrieved by sequence similarity searches (Table 6) (Additional files 4 and 5) and supported Sectm1 abundant expression in cow placenta. Moreover, RT-PCR detected Tex19.alpha but not Tex19.beta expression (Fig. 6a) which means i) that Tex19.alpha is expressed in cow placenta but it is not abundant and ii) Tex19.beta is not expressed at all in cow placenta. In rat, placenta RNA-seq and EST data were unavailable. Using RT-PCR, Tex19.1 was detected but not Tex19.2 expression (Fig. 6b). Therefore, mouse and rat exhibit similar expression patterns of Tex19 genes in placenta.

**Table 6:** Expression profile of Tex19 and Sectm1 transcripts in cow placenta using ESTs

Placenta	Tex19.alpha		Tex19.beta		Sectm1.alpha		Sectm1.beta	
EST	Count	gb_ID	Count	gb_ID	Count	gb_ID	Count	gb_ID
21,075	0	Ø	0	Ø	7	ck394172	42	Add. file 5
						aw462349		
						bp110064		
						au233908		
						au278309		
						bp108136		
						au233844		

gb_ID: genbank record identifiers Ø : empty

## Discussion

Using both molecular phylogeny and statistical test on pattern of gene copy numbers per species, we have demonstrated the co-evolution of Tex19 and Sectm1 genes. Moreover, we have shown that both gene expression patterns are characterized by opposite profiles in reproductive tissues across species. In particular, Tex19 and Sectm1 expression levels anti-correlate in primate testis which brings support to an anti-regulated gene expression. Co-evolution and anti-regulated gene expression strongly suggest a functional relationship between both genes. To determine if the cluster of co-evolving genes extends to CD7 and Uts2r, *i.e.,* Tex19 and Sectm1 neighbors on human and mouse genomes, CD7 and Uts2r accurate molecular phylogenies are required. CD7 and Uts2r phylogenies have not been reported yet. First, a CD7 protein [RefSeq:XP_007482862] and a Uts2r-like protein [RefSeq:XP_007498500] have been predicted in *Monodelphis domestica* (opossum) which is a marsupial. Second, CD7 has not duplicated in the vicinity of Sectm1 on mouse and rat genomes. Third, Uts2r has not duplicated in the vicinity of Tex19 in the same species. At first glance, CD7 and Uts2r are thus unlikely eutherian-specific and may not co-evolve with Tex19 and Sectm1. At the protein sequence level, we report the presence of 4 invariant cysteines in Tex19. In secreted proteins, invariant cysteines are frequently the signature of disulfide bridges that stabilize protein structure [48]. Immunohistochemistry experiments have established that mouse Tex19.1 protein localizes in the cytoplasm [4, 6]. Cytoplasm is a reductive environment which prevents cysteines from oxidizing into disulfide bridges [48]. Tex19 invariant cysteines are thus intriguing since cytoplasm is not expected to favor disulfide bond formation. In addition, 2 invariant cysteines of the “MCP” region are involved in a C_34_F[AT]C_37_[FY] motif which fits the fuzzier CXXC residue pattern. CXXC motifs have been reported in both redox-active catalytic enzymes, *e.g.,* thioredoxin, and zinc fingers [49, 50]. The high conservation of the “MCP” region and PSIPRED alpha-helices predictions suggest that the region might fold into a stable structural domain. Using homology modelling, we have proposed a 3D model for the Sectm1 Ig-like domain. In the model, the beta-sheets that form the classical Ig sandwich are not linked by a disulfide bridge. Instead, the disulfide is atypically predicted in the same beta-sheet. A similar structure has been previously reported in the cell surface glycoprotein CD4 which is the receptor for human immunodeficiency virus (HIV) [51]. The reason behind Tex19 and Sectm1 co-evolution is a burning question. Co-evolving genes are usually involved in the same biological process, *e.g.,* hormones and receptors. At first glance, Tex19 and Sectm1 act in distinct pathways. On one hand, expression of MMERVK10C endogenous retrovirus is up-regulated during meiosis in the mouse Tex19.1−/− testis [4, 6]. In addition, L1 retrotransposon mRNAs are derepressed in mouse Tex19.1 −/− placentas [5]. Therefore, consistent evidences suggest that Tex19 operates a control on TE activity. Expression of mouse Tex19 paralogs has been specifically detected in reproductive tissues, *i.e.,* testis, placenta, ovary and PGC [11]. Moreover, Tex19.1 deletion is associated with spermatogenesis defects and reduction of perinatal survival [6]. Tex19 is thus also involved in species reproduction. On the other hand, Sectm1 is related to the immune system. First, Sectm1 binds CD7, a 40 kDa protein of the Ig superfamily expressed by natural killer (NK) and T-cells [14]. Second, tumor cells that express Sectm1 attract monocytes [52]. Third, we proposed an Ig-like model for Sectm1 most conserved region by homology to the B cell antigen receptor. The reason why Tex19 and Sectm1 have co-evolved must thus be searched at the interface between TE activity control, species reproduction and immunity. One attractive hypothesis is that Sectm1 might trigger an immune response against cells that show TE activity while Tex19 could block the immune attack. A mechanism of intrinsic immunity against retrotransposons was previously reported [53]. Moreover, expression of endogenous retrotransposons has been associated with tissue-specific autoimmune diseases [54]. Interestingly, testis and placenta are already known to be immuno-privileged tissues [55, 56], *i.e.,* mechanisms prevent the immune system from attacking male germ cells and embryo which are both antigenic. As a perspective, immunohistochemistry experiments carried out in wild-type and Tex19 knock-out mice may prove useful to determine if the behaviour of immune system cells is altered. We are particularly interested to determine if an immune response is raised against testis and placental tissues that do not express any more Tex19 genes. Conversely, a knock-out of Sectm1 might be associated with uncontrolled expression of TE and Tex19. In our study, we showed that Tex19 and Sectm1 expression levels anti-correlate in the testis of several primates which supports anti-regulated gene expressions. Tex19 expression profile was compared in the testis of *Homo sapiens*, *Pan troglodytes*, *Pan paniscus*, *Gorilla gorilla* and *Macaca mulatta*. It is interesting to note that human, both chimpanzees and gorilla belong to the *Hominidae* family while *Macaca* belongs to the *Cercopithecidae* family. In testis, *Hominidae* show greater Tex19 expression than Sectm1 while it is the opposite in *Macaca mulatta*. It would be interesting to measure the level of expression of both genes in the testis of further Cercopithecidae primates, *e.g., Papio anubis* and *Chlorocebus sabaeus*, to determine if greater Sectm1 expression is Cercopithecidae-specific*.* However, drawing any interpretation on primate physiology based on Tex19 and Sectm1 expression profiles alone would be far-fetched. Although human Tex19 and Sectm1 are transcribed in opposite directions, both genes are separated by a 25 kb distance which is too long to support a bidirectional promoter. Indeed, bidirectional promoters are known to be short DNA sequences [57], *i.e.,* less than 1 kb. However, it is possible that Tex19 and Sectm1 genes share regulatory elements in their promoter regions and compete for transcription factor binding. Sequence analysis of both promoters is required to test this hypothesis. Moreover, both protein functions may be opposed. Antigenic cells that express Tex19 could escape the immune system. The relation between Tex19 and Sectm1 has been also striking at the protein organization level. *Haplorrhini* primates lost Tex19 C-terminal region (“iLeu” conserved region) while a Sectm1 extra C-terminal domain was acquired (conserved region B). However, Tex19 C-terminal region “iLeu” and Sectm1 extra C-terminal domain do not share any sequence similarity. Of note, human Sectm1 is expressed in a large variety of tissues, *e.g.,* colon, placenta, leukocytes, kidney and stomach and we were able to identify many tissues that express Sectm1 but not Tex19, *e.g.,* colon. Therefore, Sectm1 function does not necessarily require the co-presence of Tex19. Cells that express Sectm1 but not Tex19 may not be capable of immuno-suppression. Interestingly, we also detected Tex19 expression in human neuroblastoma, rhabdomyosarcoma and Jurkat T-cells, *i.e.,* a leukemia cell line. It is not known if those cells show TE activity and if there is a benefit for cancer cells to express Tex19. We have also demonstrated the eutherian specificity of Tex19 and Sectm1. The gene system appeared 100 million years ago in the eutherian common ancestor [58] and was transmitted to every placental mammals. We have not retrieved any species that exhibit a Sectm1 but not a Tex19 gene or the opposite except *Dasypus novemcinctus* (Xenarthra) whose genome is not complete yet. Therefore, Tex19 and Sectm1 gene system is likely to play a critical role for eutherian biology. We noticed that expression patterns of Tex19 and Sectm1 are heterogeneous across tissues and species. A striking example is placenta. Using RNA-seq data, we observed that human placenta expresses Sectm1 but not Tex19 while it is the opposite in mouse. Differences in Tex19 and Sectm1 expression may be associated with specific tuning of TE activity control across tissues and species. However, it is currently not known if TE repression and Tex19 expression level correlate.

## Conclusions

Using molecular phylogeny and statistical tests on pattern of gene copies across species, we have established the co-evolution and eutherian specificity of Tex19 and Sectm1 genes. Tex19 and Sectm1 are involved in distinct biological processes, *i.e.,* retrotransposon activity control and immunity, respectively. Sectm1 shows a broad range of tissue expression while Tex19 is specifically expressed in reproductive tissues and PGC. In addition, Tex19 and Sectm1 expression levels anti-correlate in primate testis. Our results suggest a strong functional relationship between both genes. Tex19 might suppress an immune response triggered by Sectm1 against cells that show TE activity. If the function relation between both genes could be clearly identified, new light could be shed on eutherian evolution, pathologies and biological processes as diverse as male sterility, pregnancy miscarriage, immune system evasion of tumors and auto-immune diseases.

## Additional files

Additional file 1:
**Dot plot sequence comparison between Tex19 and Sectm1 proteins.** (DOC 42 kb) Additional file 2:
**Tex19 and Sectm1 homolog detection across the 4 super-orders of eutherians.** (XLS 85 kb) Additional file 3:
**Sectm1 expression across human tissues (EST profile).** (XLS 106 kb) Additional file 4:
**Sectm1.alpha expression across cow or bull tissues (EST profile).** (XLS 68 kb) Additional file 5:
**Sectm1.beta expression across cow or bull tissues (EST profile).** (XLS 73 kb)

## Abbreviations

Tex19: Testis expressed 19
Sectm1: Secreted and transmembrane 1
ML: Maximum likelihood
TE: Transposable elements
MACS: Multiple alignment of complete protein sequences
Ig: Immunoglobulin
SP: Secretion peptide
LINE: Long interspersed nuclear element
SINE: Short interspersed nuclear element
LTR: Long terminal repeat
ERV: Endogenous retroviruses
CDS: Coding sequence
TM: Transmembrane
KO: Knock-out
HTP: High-throughput
SRA: Sequence read archive
PDB: Protein Data Bank
WGS: Whole genome shotgun
PSI-BLAST: Position specific iterated BLAST
NCBI: National center for biotechnology information
IRE: Interspersed repetitive elements
EST: Expressed sequence tag
RT-PCR: Reverse-transcription polymerase chain reaction
Uts2r: Urotensin 2 receptor
PGC: Primordial germ cells
