# Supplementary material for: Tex19 and Sectm1 concordant molecular phylogenies support co-evolution of both eutherian-specific genes
Source: BMC Evol Biol. 2015 Oct 12;15:222. doi: 10.1186/s12862-015-0506-y (PMC4603632; doi:10.1186/s12862-015-0506-y)
Supplement: Additional file 1: — Dot plot sequence comparison between Tex19 and Sectm1 proteins. (DOC 42 kb) [file 12862_2015_506_MOESM1_ESM.doc]

Dot plot sequence comparison between Tex19 and Sectm1


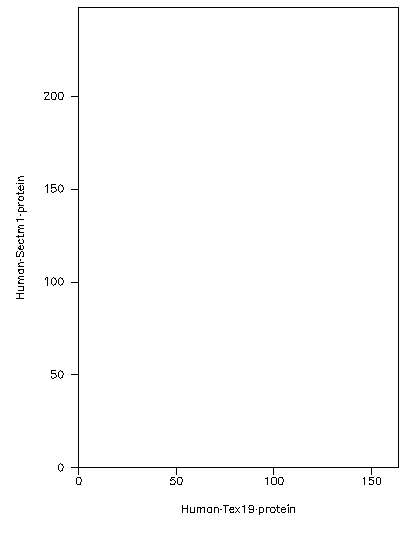


**Figure 1:** Full-length protein sequence dot plot between **human** **Tex19** (RefSeq:NP_997342) and **human** **Sectm1** (RefSeq:NP_002995). Tool: dotpath (EMBOSS), word size = 5.


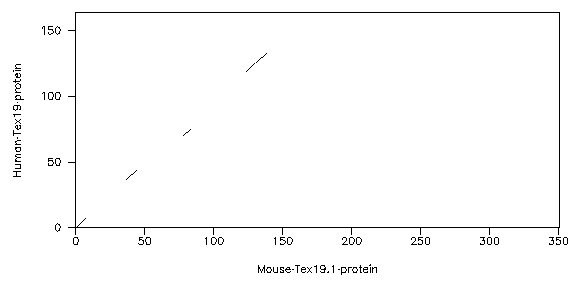


**Figure 2:** Full-length protein sequence dot plot between **human Tex19** (RefSeq:NP_997342) and **mouse Tex19.1** (NP_082878) orthologues. Tool: dotpath (EMBOSS), word size = 5.


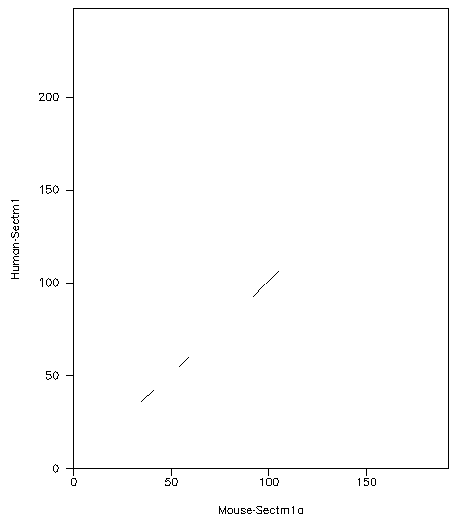


**Figure 3:** Full-length protein sequence dot plot between **human Sectm1** (RefSeq:NP_002995) and **mouse Sectm1a** (RefSeq:NP_663348) orthologues.Tool: dotpath (EMBOSS), word size = 5.
